# Supplementary material for: Cell freezing protocol suitable for ATAC-Seq on motor neurons derived from human induced pluripotent stem cells
Source: Sci Rep. 2016 May 5;6:25474. doi: 10.1038/srep25474 (PMC4857123; doi:10.1038/srep25474)
Supplement: Supplementary Information [file srep25474-s1.pdf]

Supplementary Materials

Cell freezing protocol suitable for ATAC-Seq on motor neurons derived from human induced pluripotent stem cells

Pamela Milani[1], Renan Escalante-Chong[1], Brandon C. Shelley[2], Natasha L. Patel-Murray[1], Xiaofeng Xin[1], Miriam Adam[1], Berhan Mandefro[2,3], Dhruv Sareen[2,3,4], Clive N. Svendsen[2,3,4], Ernest Fraenkel[1]*

[1]*Department of Biological Engineering, Massachusetts Institute of Technology, Cambridge, Massachusetts, 02139, United States of America*

[2]*Board of Governors-Regenerative Medicine Institute, Cedars-Sinai Medical Center, Los Angeles, California, 90048, United States of America*

[3]*iPSC Core, The David and Janet Polak Foundation Stem Cell Core Laboratory, California, 90048, United States of America*

[4]*Department of Biomedical Sciences, Cedars-Sinai Medical Center, Los Angeles, California, 90048, United States of America*

[*] *fraenkel-admin@mit.edu*

Supplementary Tables

Table S1. Information about the number of cells used for the experiment, the percentage of cell death assessed by chromatin condensation and the number of nuclei recovered from cryopreserved (C) neurons.

Sample	# of cells	Cell death (%)	# of recovered nuclei
**C1**	947,150	11.0	762,000
**C2**	1,282,500	8.3	921,000
**C3**	1,225,500	12.3	849,000

Table S2. Mitochondrial DNA (mtDNA) contamination in fresh (F) and cryopreserved (C) iMNs.

Sample	mtDNA (%)
**F1**	32.14
**F2**	27.97
**F3**	31.44
**C1**	45.33
**C2**	49.61
**C3**	47.69

Table S3. Sequences of the primers used to amplify open-chromatin and gene desert regions.

Primer ID	Primer sequence
**GAPDH gene promoter Fw**	CATCTCAGTCGTTCCCAAAGT
**GAPDH gene promoter Rv**	TTCCCAGGACTGGACTGT
**Gene desert region Fw**	AACTGGCTAGTAAGGAGTGAATG
**Gene desert region Rv**	GGGAATGGAAAGAAGTCCACTAT
**B2M gene promoter Fw**	GGAAAGTCCCTCTCTCTAACCT
**B2M gene promoter Rv**	GCGACGCCTCCACTTATATT
**Gene desert region #2 Fw**	CCCAAACTCTGAGAGGCTTATT
**Gene desert region #2 Rv**	GAGCCATCATCTAGACACCTTC

Supplementary Figures

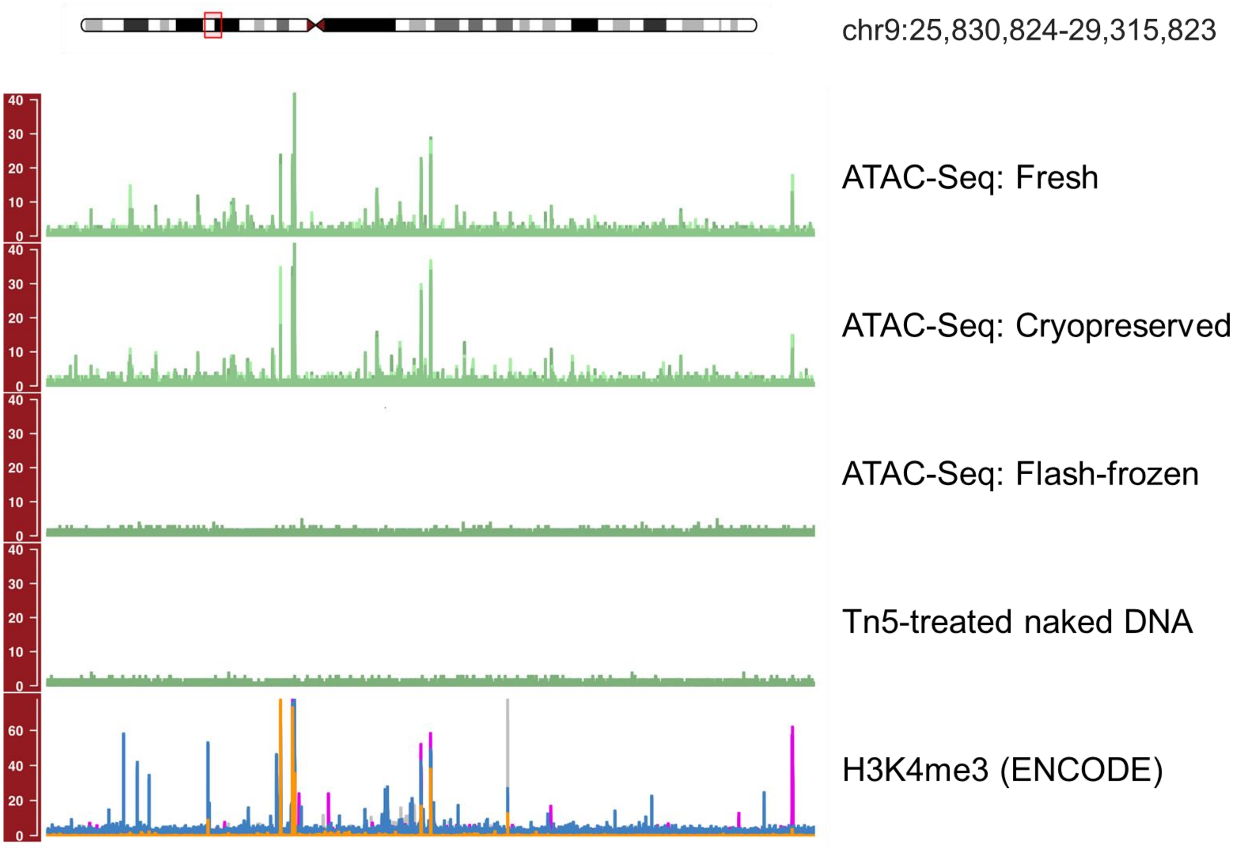

Figure S1. ATAC-Seq tracks of a large genomic region (3.5 Mbp). The tracks were visualized with the *Gviz* package: peaks from both fresh and cryopreserved neurons were sharp and overlapped with H3K4me3 ChIP-Seq peaks from ENCODE; the reads from flash-frozen neurons were distributed noisily across the genome (F = fresh, FF = flash-frozen, C = cryopreserved).

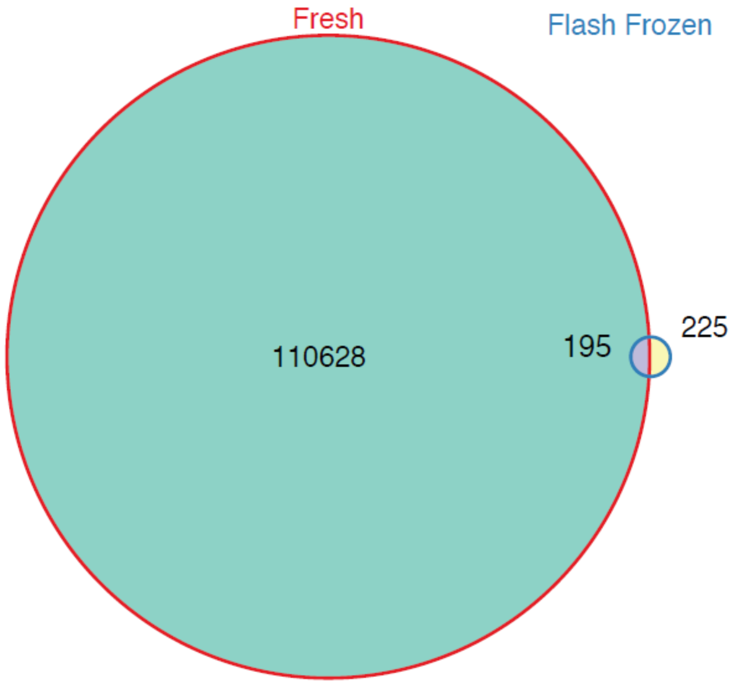

Figure S2. Venn diagram showing the overlap of the peaks between fresh and flash-frozen iMNs. The reads from the three technical replicates from the fresh iMNs were merged before calling the peaks with MACS2 and calculating the overlap with the peaks from flash-frozen iMNs. We observed that 236 out of 461 peaks detected in the flash-frozen iMNs overlapped with the peaks obtained from the fresh cells. In some cases, multiple peaks from a sample mapped to a single peak in the second sample. Such ties were counted as a single overlap, resulting in the 195 overlapping peaks displayed on the Venn diagram.

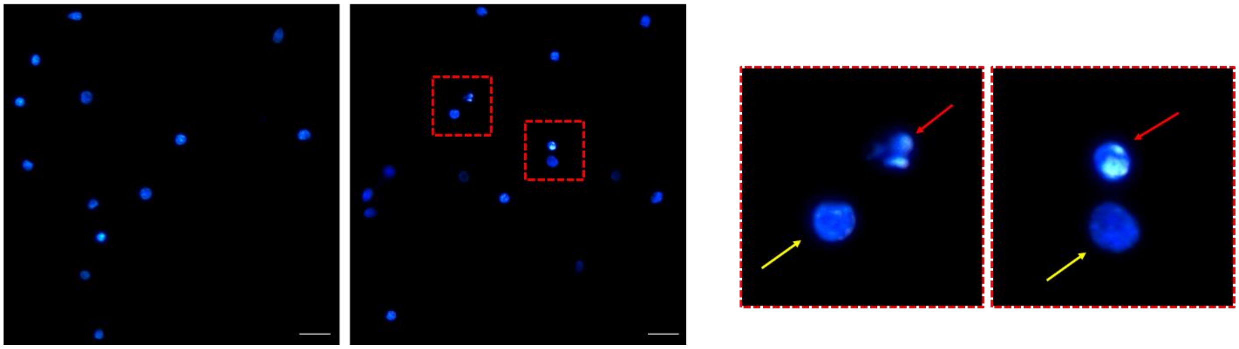

Figure S3. Two representative microscopic pictures of thawed cells stained with Hoechst 33342 for the assessment of neuronal death based on chromatin condensation. The right panel shows the corresponding enlarged images from the left panel. The red arrows indicate apoptotic cells with condensed and fragmented chromatin and bright Hoechst signal, while the yellow arrows indicate viable cells with diffuse staining. Scale bar = 40 µm.

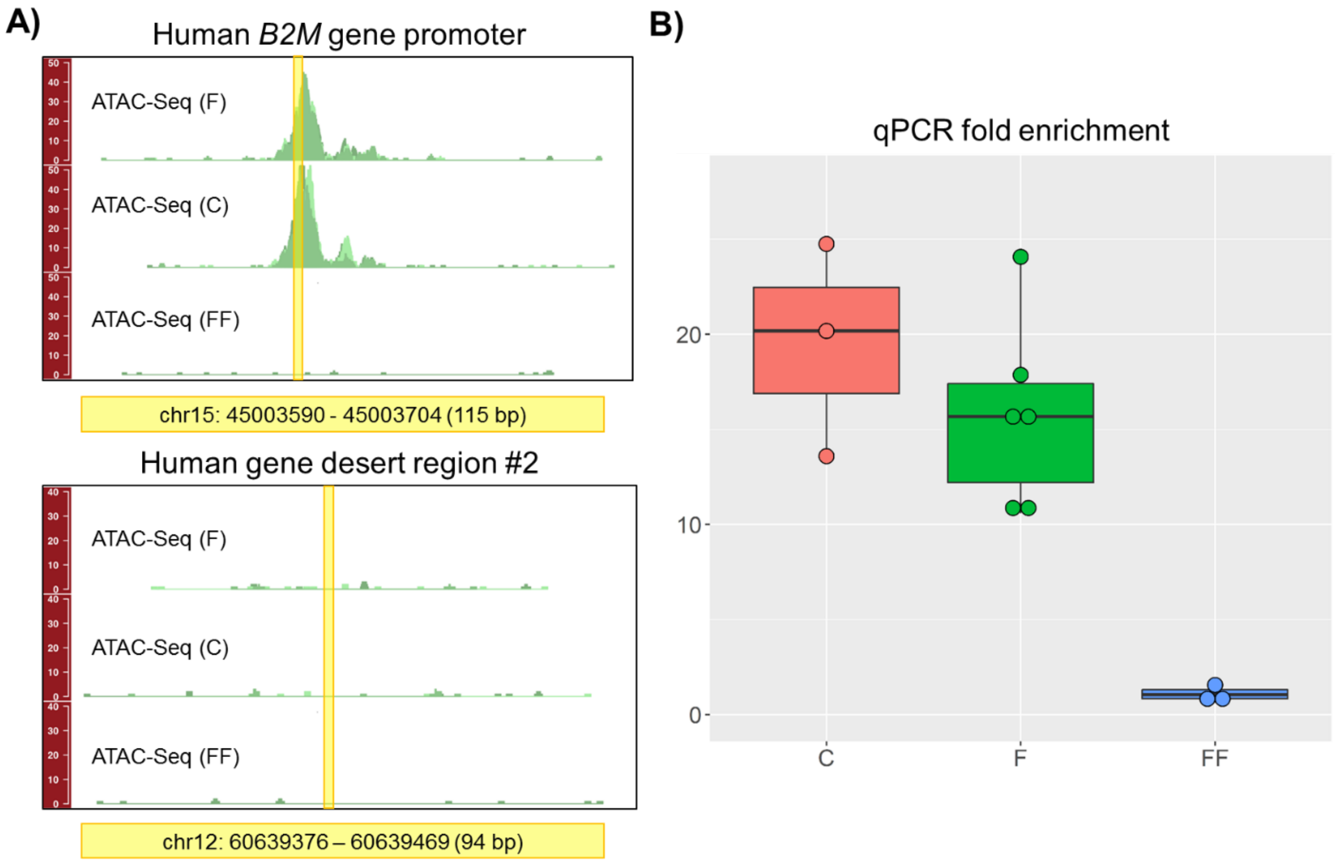

Figure S4. Real-time qPCR for the assessment of the quality of ATAC-Seq libraries.

(A) Genomic locations of the primers used to amplify positive (human *B2M* gene promoter) and negative (human gene desert region) control sites. (B) Fold enrichment of the open-chromatin site over the Tn5-insensitive site: while real-time qPCR experiments showed high enrichment for fresh and cryopreserved samples, poor results were obtained with flash-frozen cells (F = fresh, FF = flash-frozen, C = cryopreserved).

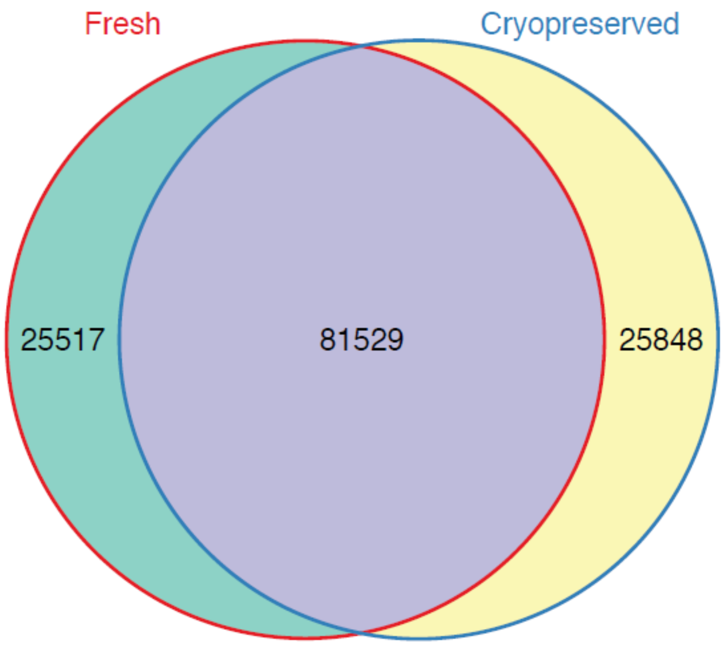

Figure S5. Venn diagram showing the overlap of the peaks between fresh and cryopreserved iMNs. The reads from the three technical replicates from both fresh and cryopreserved iMNs were merged before calling the peaks with MACS2 and calculating the overlap between the two conditions.
